# Supplementary material for: Associations of lifetime concussion history and repetitive head impact exposure with resting-state functional connectivity in former collegiate American football players: An NCAA 15-year follow-up study
Source: PLoS One. 2022 Sep 9;17(9):e0273918. doi: 10.1371/journal.pone.0273918 (PMC9462826; doi:10.1371/journal.pone.0273918)
Supplement: S1 File — Linear regressions for the effect of individual covariates on each of the resting-state functional connectivity (rsFC) outcomes described in body of the manuscript. (DOCX) [file pone.0273918.s001.docx]

**S1 File**

**Supplemental Results:**

**Linear regressions for the effect of individual covariates on each of the resting-state functional connectivity (rsFC) outcomes described in body of the manuscript.**

Highlighted results indicate a statistically significant univariable association where the 95% confidence interval for the unstandardized beta value did not include ‘0’, and the associated *p*-value was < 0.05. Unstandardized B-values are in units of Pearson *r* correlation values representing the average rsFC between a set of functional regions of interest [ROIs] in the brain.

Body mass index (BMI) and MRI acquisition site were observed to have at least one significant univariable association and were each therefore included as covariates in all of the multivariable models testing for the associations between lifetime concussion history and repetitive head impact exposure on rsFC measures.

**Age**

- Whole-brain – unstandardized Β = 0.000, 95% CI = -0.002, 0.001), *p* = .89
- Within
  - DMN – unstandardized Β = -0.001, 95% CI = -0.009, 0.008), *p* = .47
  - DAN – unstandardized Β = 0.000, 95% CI = -0.015, 0.014), *p* = .96
  - FPCN – unstandardized Β = 0.002, 95% CI = -0.009, 0.012), *p* = .77
- Between
  - DMN-DAN – unstandardized Β = 0.001, 95% CI = -0.007, 0.010), *p* = .75
  - DMN-FPCN – unstandardized Β = 0.004, 95% CI = -0.004, 0.000), *p* = .31
  - DAN-FPCN – unstandardized Β = -0.002, 95% CI = -0.011, 0.006), *p* = .58

**BMI**

- Whole-brain – unstandardized Β = 0.000, 95% CI = 0.000, 0.001), *p* = .55
- Within
  - DMN – unstandardized Β = -0.001, 95% CI = -0.004, 0.002), *p* = .59
  - DAN – unstandardized Β = 0.000, 95% CI = -0.005, 0.005), *p* = .93
  - FPCN – unstandardized Β = -0.003, 95% CI = -0.007, 0.000), *p* = .08
- Between
  - DMN-DAN – unstandardized Β = -0.001, 95% CI = -0.004, 0.002), *p* = .71
  - DMN-FPCN – unstandardized Β = -0.001, 95% CI = -0.004, 0.002), *p* = .49
  - DAN-FPCN – unstandardized Β = 0.004, 95% CI = 0.001, 0.007), *p* = .01

**MRI acquisition site**

- Whole-brain – unstandardized Β = -0.006, 95% CI = -0.010, -0.002), *p* = .003
- Within
  - DMN – unstandardized Β = -0.027, 95% CI = -0.058, 0.003), *p* = .08
  - DAN – unstandardized Β = -0.059, 95% CI = -0.108, -0.010), *p* = .02
  - FPCN – unstandardized Β = -0.017, 95% CI = -0.056, 0.021), *p* = .37
- Between
  - DMN-DAN – unstandardized Β = 0.028, 95% CI = -0.003, 0.058), *p* = .08
  - DMN-FPCN – unstandardized Β = 0.028, 95% CI = 0.000, 0.057), *p* = .048
  - DAN-FPCN – unstandardized Β = -0.037, 95% CI = -0.067, -0.007), *p* = .02

**Post hoc power analyses for each multivariable linear regression model included in the paper.**

|  | **“High” vs. “Low” Concussion History** | | **4-Category Concussion History** | |
| --- | --- | --- | --- | --- |
| **Self-Reported Concussion History** | | | | |
| **Outcome** | **Adjusted R^2^** | **Power estimate**  **1-β** | **Adjusted R^2^** | **Power estimate** |
| **Whole-brain** | .120 | .735 | .110 | .734 |
| **Within-network** |  |  |  |  |
| DMN | .000 | .246 | .009 | .265 |
| DAN | .066 | .841 | .087 | .901 |
| FPCN | .078 | .824 | .073 | .766 |
| **Between-network** |  |  |  |  |
| DMN-DAN | .012 | .378 | .043 | .519 |
| DMN-FPCN | .028 | .549 | .003 | .487 |
| DAN-FPCN | .137 | .911 | .150 | .905 |
